# Native mass spectrometry analyses of chaperonin complex TRiC/CCT reveal subunit N-terminal processing and re-association patterns

**DOI:** 10.1038/s41598-021-91086-6

**Published:** 2021-06-22

**Authors:** Miranda P. Collier, Karen Betancourt Moreira, Kathy H. Li, Yu-Chan Chen, Daniel Itzhak, Rahul Samant, Alexander Leitner, Alma Burlingame, Judith Frydman

**Affiliations:** 1 Department of Biology, Stanford University, Stanford, CA USA; 2 Department of Chemistry, University of California San Francisco, San Francisco, CA USA; 3 Chan-Zuckerberg BioHub, San Francisco, CA USA; 4 Department of Biology, Institute of Molecular Systems Biology, Zurich, Switzerland

**Keywords:** Biochemistry, Biophysics

## Abstract

The eukaryotic chaperonin TRiC/CCT is a large ATP-dependent complex essential for cellular protein folding. Its subunit arrangement into two stacked eight-membered hetero-oligomeric rings is conserved from yeast to man. A recent breakthrough enables production of functional human TRiC (hTRiC) from insect cells. Here, we apply a suite of mass spectrometry techniques to characterize recombinant hTRiC. We find all subunits CCT1-8 are N-terminally processed by combinations of methionine excision and acetylation observed in native human TRiC. Dissociation by organic solvents yields primarily monomeric subunits with a small population of CCT dimers. Notably, some dimers feature non-canonical inter-subunit contacts absent in the initial hTRiC. This indicates individual CCT monomers can promiscuously re-assemble into dimers, and lack the information to assume the specific interface pairings in the holocomplex. CCT5 is consistently the most stable subunit and engages in the greatest number of non-canonical dimer pairings. These findings confirm physiologically relevant post-translational processing and function of recombinant hTRiC and offer quantitative insight into the relative stabilities of TRiC subunits and interfaces, a key step toward reconstructing its assembly mechanism. Our results also highlight the importance of assigning contacts identified by native mass spectrometry after solution dissociation as canonical or non-canonical when investigating multimeric assemblies.

## Introduction

**Figure 1:**
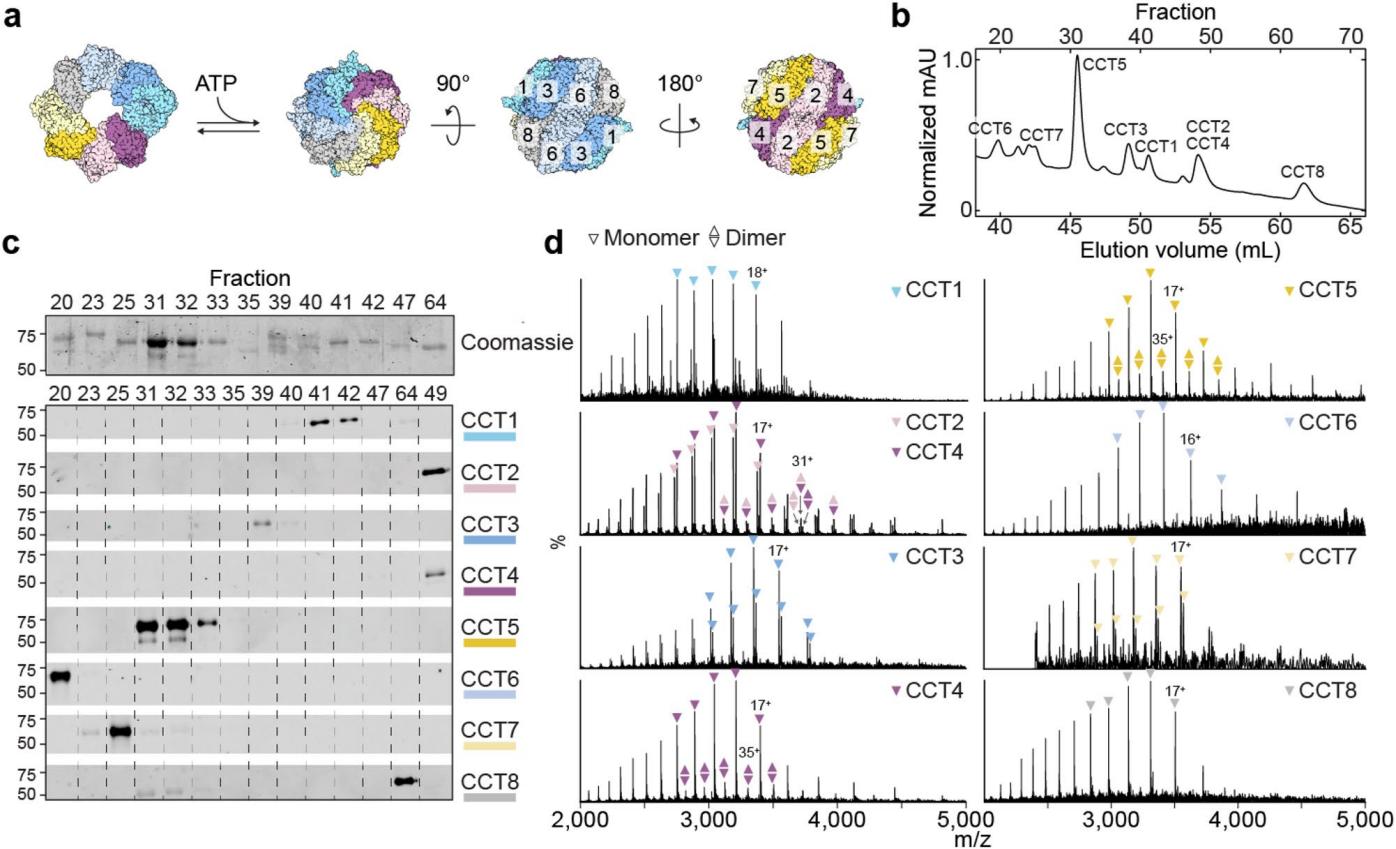
hTRiC subunits can be chromatographically separated for downstream intact analysis. (**a**) Top and side views of the TRiC hexadecamer, showing subunit arrangement within the two antiparallel rings. Cycles of ATP hydrolysis catalyse ring opening and closure. (**b**) Reverse phase chromatogram of recombinant hTRiC, with peaks labelled by the predominant eluting subunit(s), as determined by Western blots shown in (**c**). (**d**) Intact, denatured mass spectra of reverse phase fractions containing each hTRiC subunit. For each assigned subunit mass, five peaks are annotated with triangles and one peak with its charge state. Fractions containing CCT5, CCT2, or CCT4 contain minor populations of masses corresponding to dimers, designated by two triangles annotated over five odd charge states. (Even charge states are not labelled with the double triangle notation for simplicity, but can be inferred to overlap with monomer peaks.) In the CCT2/CCT4 spectrum, CCT2 and CCT4 homodimers were detected as well as CCT2-CCT4 heterodimer.

The eukaryotic chaperonin TRiC (TCP1-ring complex, or CCT, cytosolic chaperonin containing TCP1) is an essential protein complex that assists in folding and preventing aggregation of approximately 10% of the proteome, including obligate substrates actin and tubulin^1–3^. Eukaryotic TRiC is a 1 MDa hexadecamer, comprising two antiparallel rings of eight paralogous subunits (CCT1-8, or CCTα, β, γ, δ, ε, ζ, η, θ) surrounding a central chamber (Fig. 1a). Each ring contains two hemispheres with higher and lower ATP affinity, creating a gradient of ATP hydrolysis that catalyses the conformational cycle of substrate folding and release^4,5^. Thus, correct assembly of the specific subunit arrangement of the TRiC complex is critical. Assembly does not occur spontaneously ex vivo, and how the cell accomplishes the task remains unclear.

Initial insight into the structure of TRiC relied on its purification from bovine and mouse testes^6,7^, and from yeast^8^. While tags, substrates, and cofactors aided in purifying and stabilizing these types of preparations for structural and biochemical studies^9^, their intrinsic heterogeneity and low yields precluded certain applications, and may have contributed to some ambiguity in reported arrangements^1^. The development of a protocol to express and purify correctly assembled and functional recombinant human TRiC (herein hTRiC) from insect (*Trichoplusia ni*) cells has facilitated structural studies and the exploration of new functional avenues^10^. However, the extent of post-translational processing, in addition to the amounts and identities of co-purifying proteins, in standard preparations of recombinant hTRiC have not been described.

Here, we employ a range of mass spectrometry (MS)-based analytical approaches to characterize the composition, stability, and processing of insect cell-derived hTRiC. We find that subunits are N-terminally processed, including methionine cleavage and acetylation, according to enzymatic regimes that are conserved in human cells. We identify and roughly quantify trace co-purifiers in the existing purification pipeline, including *T. ni* TRiC subunits and substrates. Lastly, we use native mass spectrometry to study the dissociation pathway of hTRiC. We show that following chemical disruption of the 16-mer, subunits can engage in re-formed canonical and non-canonical contacts; and we discuss potential implications for chaperonin assembly. These findings provide a resource and foundation for future exploration of TRiC assembly and function.

## Results

### Chromatographic separation enables mass analysis of intact subunits

We purified human TRiC by co-expressing CCT1-CCT8 as described previously^10^. CCT1 contained an internal CBP (calmodulin-binding peptide) tag to enable an affinity purification step. Negative stain electron microscopy images validated that the proteins assembled into the expected stacked double rings and cross-linking mass spectrometry confirmed the recombinant complex adopts the expected canonical subunit arrangement (Supplementary Fig. 1; Supplementary Data 1)^10^. ATP-dependent chaperonin functionality was confirmed through lid closure assays and its ability to promote folding of actin, an obligate TRiC substrate (Supplementary Fig. 1). Next, we wished to recover individual subunits for downstream analysis. We separated the subunits by reverse phase chromatography under denaturing conditions (Fig. 1b). This yielded fractions containing the isolated subunits, as confirmed by Coomassie stain and Western blot (Fig. 1c). The most recalcitrant to separation from each other were adjoining subunits CCT2 and CCT4, as observed previously in similar reverse phase analyses of bovine TRiC^4^. CCT5 was recovered in the greatest abundance (Fig. 1c), despite equimolar stoichiometry of all subunits in the assembled TRiC sample prior to separation (Supplementary Fig. 1).

To measure masses of the CCT subunits, fractions were manually injected into a mass spectrometer equipped for transmission of intact proteins. The resulting mass spectra (Fig. 1d) featured dominant charge state distributions characteristic of partially unfolded monomers, which were used to derive masses (Supplementary Data 2). A predominantly-CCT4 fraction was used in addition to the joint CCT2-CCT4 fraction to distinguish between CCT2 and CCT4. Fractions containing CCT5, CCT2, and CCT4 also contained charge series with masses matching partially unfolded dimers (Fig. 1d; Supplementary Data 4). To support the accuracy of the masses with an orthogonal measurement, we ran a similar gradient by LC–MS, with direct injection and continual recording of mass spectra on a different instrument. The same masses were recorded in the same order of subunit elution, with high charge states indicating complete rather than partial unfolding (Supplementary Fig. 2).

### hTRiC co-purifies with trace amounts of insect cell homologs and substrate

**Figure 2:**
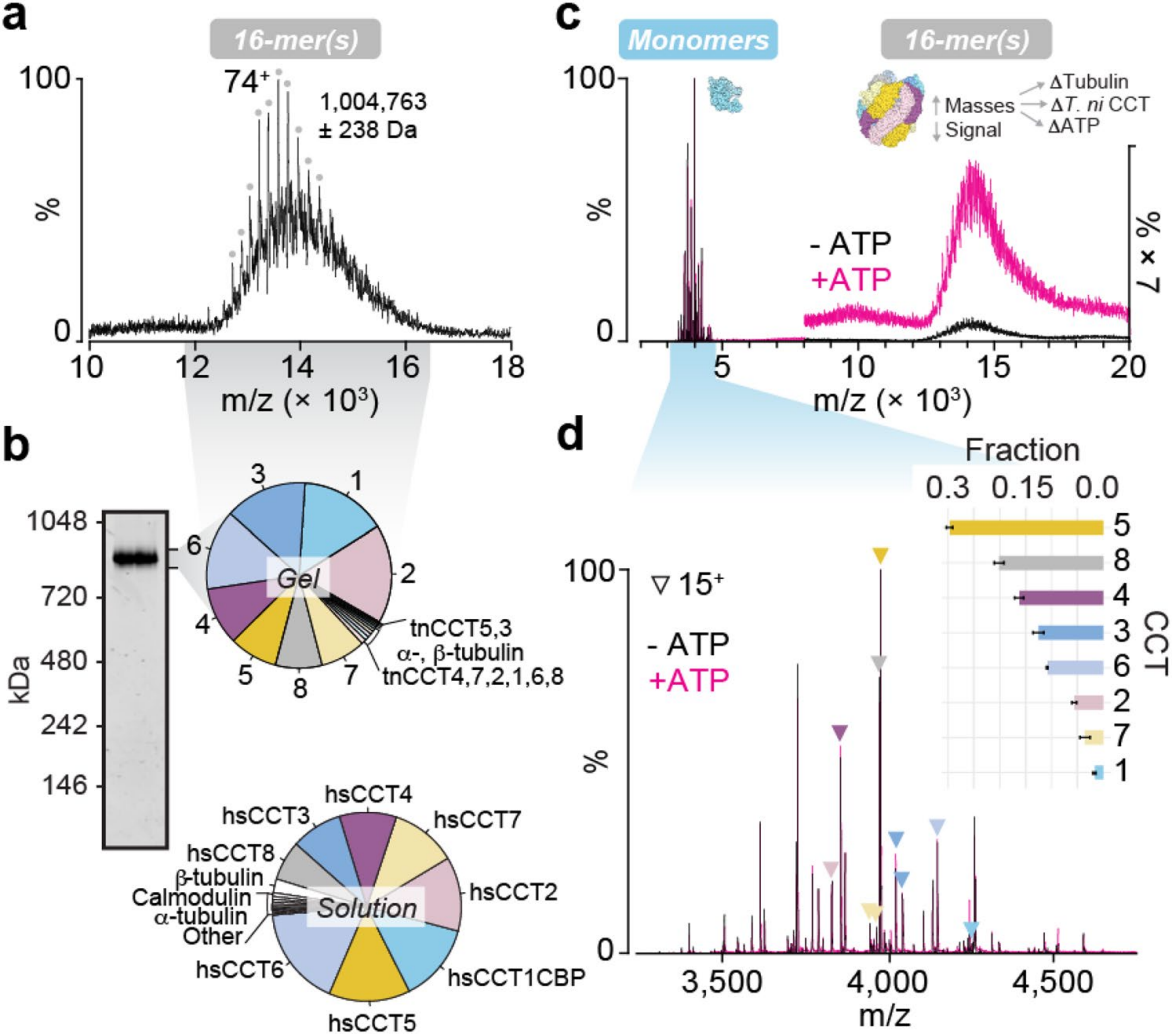
The hTRiC holocomplex is heterogeneous in mass and stabilized by ATP. (**a**) High m/z region of a native mass spectrum of TRiC, with optimal transmission of the complex. The labelled mass, calculated using the charge series designated by gray circles, corresponds closely to a 16-mer associated with tubulin and ATP. (**b**) Proteomic analysis of hTRiC solution (bottom) and the hTRiC complex excised as a band from NativePAGE (top). Pie charts show filtered hits in order of relative abundances estimated by label-free intensity-based quantification. (**c**) Complete native mass spectra of hTRiC before and after addition of ATP, with instrument conditions favoring transmission of the monomers. Signal to noise of the 16-mer is low, but improves upon addition of ATP, indicating stabilization or mass coalescence. (**d**) Detailed view of the regions of native MS in *c* containing dissociated CCT subunits. Triangles indicate each 15^+^ charge state. The inset bar graph shows each subunit’s fractional abundance relative to all measured monomers; i.e., CCT5 comprised 30% of all monomer signal. More abundant subunits are more stable after dissociating from the 16-mer, remaining folded for the duration of the experiment. Bars show the average and SD between + /− 500 µM ATP (black and pink mass spectra) to emphasize that addition of ATP, while it stabilized the complex, did not affect the relative abundances of detected monomers.

We next analysed hTRiC by native mass spectrometry, using an instrument capable of very high mass transmission^11^. We reasoned that this approach should highlight any mass shifts caused by the prior denaturing conditions (e.g. due to loss of bound ligands) and offer insight into the purity of the assembled complex. The 16-mer was detected, but with poor signal to noise and a high baseline of broad peaks, indicating mass heterogeneity (Fig. 2a). A species could be assigned weighing just over 1 MDa. Based on our measured subunit masses (Supplementary Data 2), a double ring containing only the 16 CCT subunits would have mass 950 kDa (958 kDa with 16 ATP bound). This discrepancy is unlikely to be entirely due to residual solvation, and indicates the possible presence of substrates or cofactors. To identify them, we performed in-solution and in-gel enzymatic digests of hTRiC and subjected these to standard bottom-up proteomic analysis. Intensity-based absolute quantification (iBAQ) was used to estimate relative protein abundances in solution versus only in the region of a native SDS-PAGE gel containing the intact 16-mer (Fig. 2b; Supplementary Data 3). The human TRiC subunits were the eight most abundant proteins in both samples, comprising over 93% of the total protein in solution and over 95% in the excised gel sample. Small amounts of all eight insect cell TRiC subunits were also present, as were insect cell chaperones Hsp70 and Hsp90 (Supplementary Data 3﻿). Tubulin, an obligate substrate of TRiC^12^, was also detected in both α- and β- forms, comprising ~ 3% of the protein in solution and migrating with TRiC in the native gel (Fig. 2b; Supplementary Data 3). Whereas α-tubulin levels in solution and in the complex were similar, β-tubulin was substantially less abundant in the excised complex. These co-purifiers were also present in hTRiC purified using a different approach, and containing a GFP-tagged CCT6 instead of CBP-tagged CCT1 (Supplementary Data 3). Other trace proteins that appeared in multiple datasets included mitochondrial ADP/ATP carrier; and translation elongation factors which are known TRiC substrates (Supplementary Data 3)^3^.

### Recombinant CCT N-termini are processed by methionine excision and acetylation

Contrasting the broadened signal of the complex at high m/z, peaks also appeared in native mass spectra between 3000 and 5000 m/z that could be sharply resolved (Fig. 2c). The corresponding masses matched those measured for hTRiC subunits (Supplementary Data 2), and the charge states indicated folded monomers. Thus, these were native-like CCT subunits which must have dissociated from complexes, possibly during buffer exchange or ionization. Monomer peaks did not shift with addition of ATP, although ATP did increase the signal intensity of the high-mass region, suggesting that ATP preferentially bound the assembled TRiC but not free subunits (Fig. 2c). We assigned the spectra and deconvolved relative abundances of subunits, a proxy for their stability in the folded state following dissociation. Similar to the yield after reverse phase chromatography (Fig. 1c), CCT5 was measured at the highest intensity; followed by CCT8 and CCT4 (Fig. 2d). Present at the lowest levels were CCT1 and CCT7.

**Figure 3:**
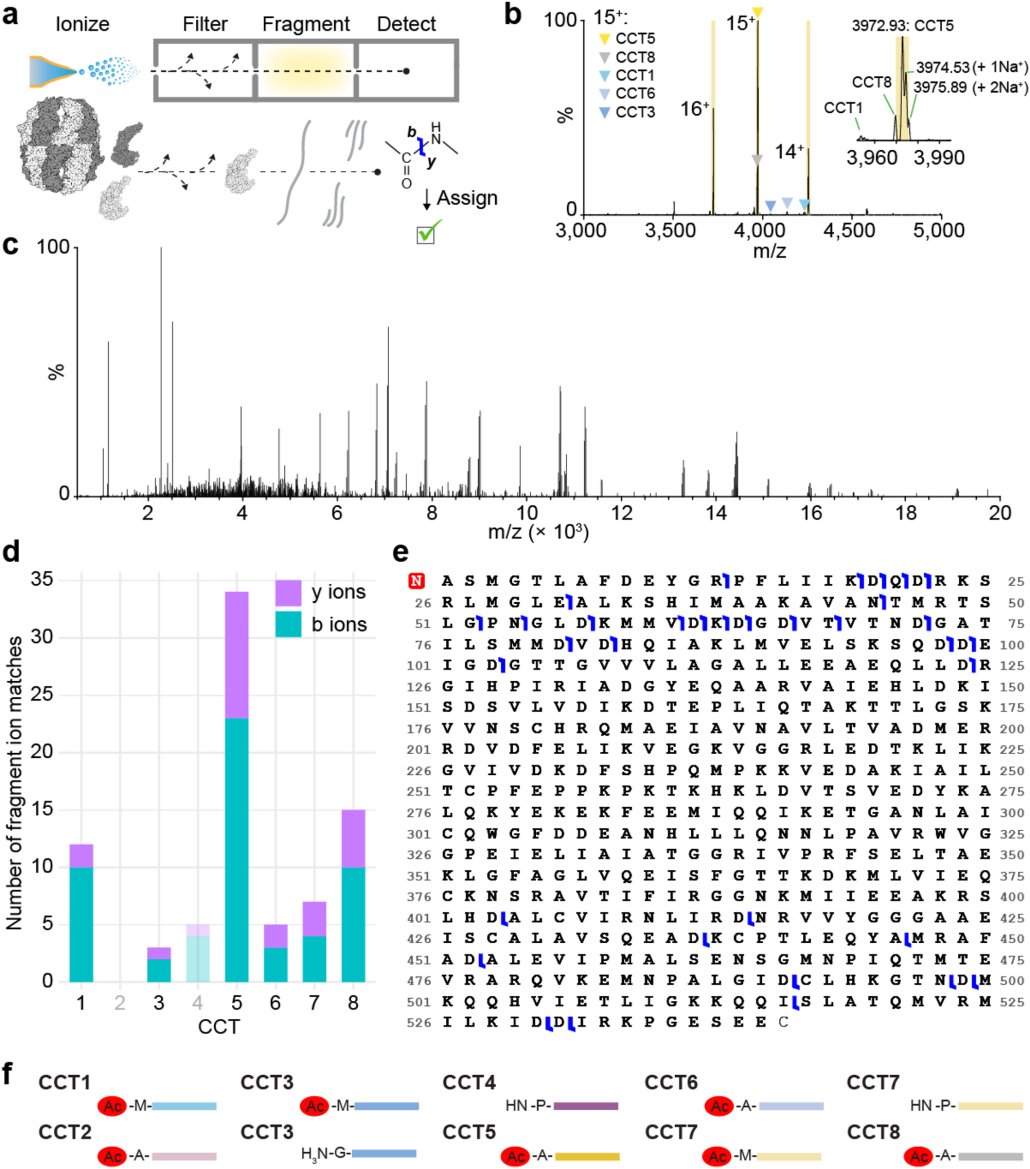
CCT5 is N-terminally processed by methionine excision and acetylation. (**a**) Schematic of the top-down MS experiment. The chaperonin is color-coded as though subunit identities are unknown. (**b**) Monomer region of the native mass spectrum of a hTRiC variant lacking subunits CCT2 and CCT4, containing predominantly CCT5 and trace amounts of other subunits (colored triangles). Each highlighted peak was fragmented. The inset is a magnified view of the most abundant charge state, 15^+^, showing that the resolution is high enough to distinguish sodium adducts. The CCT8 15^+^ peak is just 3 m/z to the left of the peak of interest. (**c**) Deisotoped peptide spectrum resulting from fragmentation of the 15^+^ peak in *b*. (**d**) Numbers of fragment ion matches from all highlighted peaks in **b** to sequences of all CCT subunits, divided into b- and y-ions. CCT2 and CCT4 are faded because they were not present in the sample, and can therefore serve as negative controls for the expected number of false positives. (**e**) Primary sequence of human CCT5 without the initiator methionine. Blue marks indicate at least one reported peptide mass (in data from all three highlighted peaks in **b**) supporting an N- or C-terminal ion cleaved at the given site. The red *N* at the N-terminus designates acetylation. (**f**) Measured and inferred N-terminal states of post-translationally processed recombinant hTRiC subunits expressed in insect cells. Red ovals denote acetylation.

The masses we measured did not exactly match predicted masses based on subunit amino acid sequences, suggesting post-translational modification. A likely candidate site is the N-terminus, which is modified on the majority of polypeptides in eukaryotes, often cotranslationally^13^. To support modification at the N-terminus directly, we turned to top-down mass spectrometry. This approach builds on native MS by isolating individual ion species in *m/*z and subjecting them to high energy collisional dissociation to break peptide bonds, generating b- and y-ion fragments that can be mapped to an amino acid sequence (Fig. 3a)^14^. We used a hTRiC variant lacking CCT2 and CCT4 because the monomer region of its native mass spectrum contained fewer peaks than the crowded WT monomer region, making it more likely that peptides resulting from fragmentation would derive from a single parent ion (Fig. 3b); and because CCT2 and CCT4 could be used as negative controls for peptide matching. Three charge states from the same monomer series, believed to be CCT5, were isolated and fragmented into peptides (Fig. 3c). We found many more matches between the derivative peptide masses and the CCT5 sequence than any other subunit, further confirming its identity, which had previously relied on antibody specificity (Fig. 3d). The second most supported assignment was CCT8 followed by CCT1. We suspect these are due partially to true peptide hits from imperfect peak isolation and trace ion carry-over into the MS isolation windows, as CCT8 is the closest in mass to CCT5 meaning its peaks appear at only slightly lower m/z than CCT5, and CCT1 peaks are not far to the left of CCT8 (Fig. 3b). The few remaining peptide matches to CCT sequences are likely primarily false positives, similar to the number of matches for CCT4, which was not present in the sample (Fig. 3d). Because peptides generated by this method of top-down fragmentation derive from the termini which are the least conserved regions of the CCT paralogs^15^, false positives due to sequence similarity were not a concern. The data supported N-terminal methionine excision and acetylation of CCT5: satisfactory sequence coverage of the N-terminus was observed only when these modifications were introduced into the theoretical fragmented polypeptide (Fig. 3e).

We next checked the remaining subunit mass measurements against their sequences to derive modifications. The most parsimonious assignments are shown in Fig. 3f and listed in Supplementary Data 2. Masses are consistent with N-terminal methionine cleavage from CCT2, CCT4, CCT5, CCT6, and CCT8, as well as with loss of two methionines from partial populations of CCT3 and CCT7. Masses also indicate N-terminal acetylation except for those subunits where the residue exposed after methionine removal is proline (CCT4; one of two CCT7 states) or glycine (one of two CCT3 states).

### Solvent disruption of the hTRiC 16-mer precedes widespread dimerization

When all CCT subunits are co-expressed together they assemble in a TRiC complex that follows the canonical arrangement^1^. However, combining independently expressed subunits ex vivo does not result in a homogeneous complex with the proper arrangement, raising the question of how the complex assembles and whether subunits contain the specificity information to engage in the correct contacts^16^. In an effort to infer the organization and assembly pathway of the complex from (dis)assembly intermediates, previous studies tried to examine TRiC subcomplexes present in cell lysates by combined native gel analysis and immunoblotting^7,17^. To capture novel intermediates, we disrupted assembled hTRiC complexes by addition of 25% organic solvent (dimethyl sulfoxide, DMSO, or methanol) immediately prior to analysis by native mass spectrometry. This approach does not fully denature proteins and has been used previously to yield insight into complex formation and topology^18–22^.

**Figure 4:**
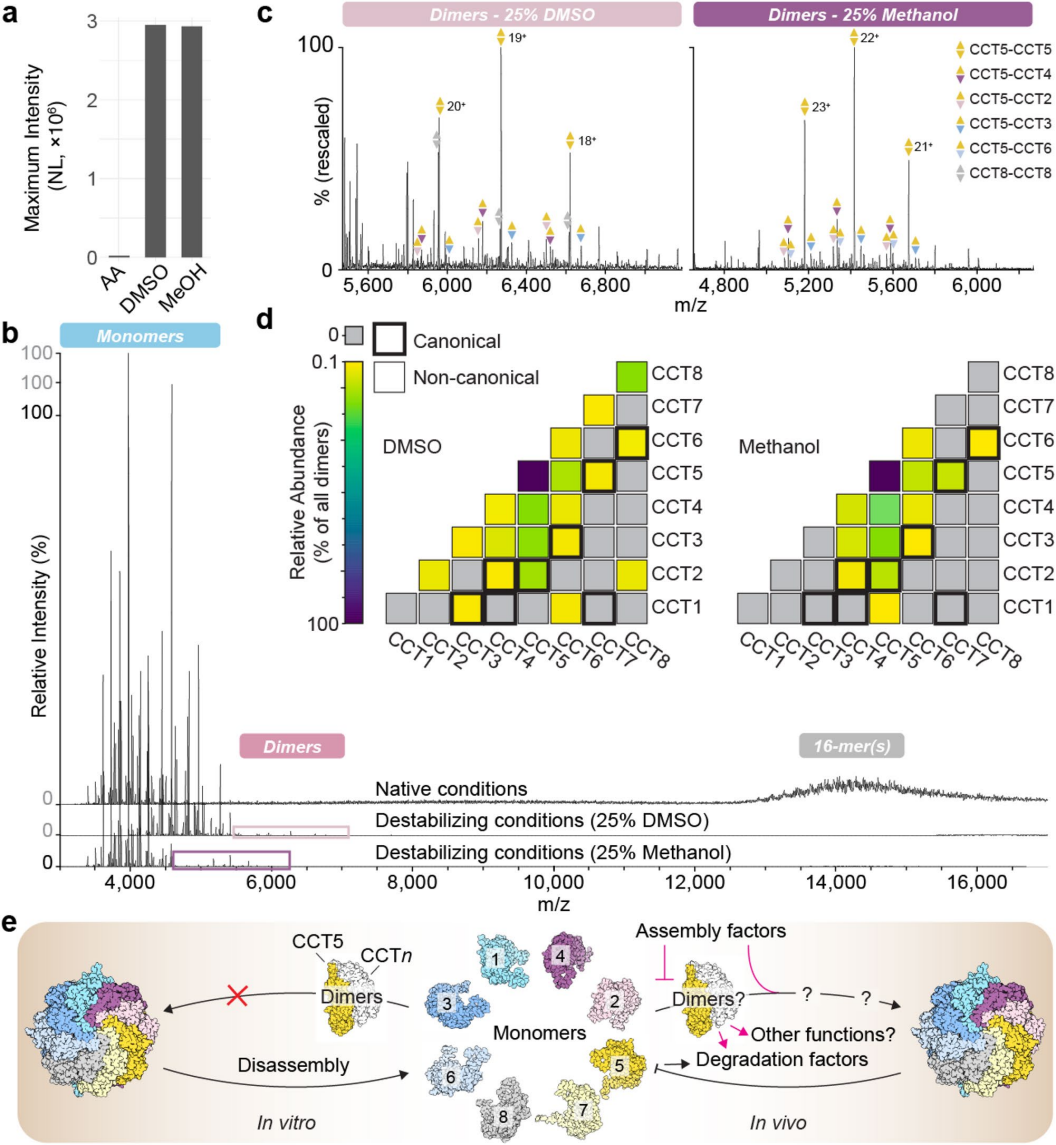
Chemical disruption of the hTRiC 16-mer enables subsequent dimerization. (**a**) The monomer signal intensity of the same hTRiC sample increases over 100-fold upon addition of 25% DMSO or methanol (MeOH) (NL = Normalized Level). This supports the original presence of predominantly intact 16-mers and their thorough dissociation into monomers. (**b**) Native mass spectra of intact and solution-dissociated hTRiC. Monomer, dimer, and 16-mer regions of signal are observed. (**c**) Detail view of the dimer regions in b.﻿ Peaks are sufficiently resolved to assign dimers unambiguously; three charge states for the five most abundant assigned dimers are annotated in each mass spectrum. (**d**) Relative abundances of all assigned CCT dimers after DMSO or methanol dissociation. Squares with a bold outline represent interfaces that exist in correctly assembled TRiC. The underlying data and quantification of each subunit’s contribution to the total dimer intensity is provided in Supplementary Data 4. (**e**) Schematic of TRiC assembly and dissociation in vitro and in vivo, where CCT5 is posited to play a key role in processes involving dimers.

Upon addition of DMSO or methanol, the signal intensity of the monomers in the native mass spectrum increased over 100-fold, bringing the monomer concentration from nanomolar to low micromolar (Fig. 4a). This demonstrates successful disassembly of a sample consisting predominantly of higher-order hetero-oligomers in the mass spectrometry compatible buffer. DMSO lowered all charge state distributions, shifting peaks to the right in *m/z* (Fig. 4b). We did not see evidence of subunit unfolding, which would have yielded higher charge state distributions; meaning any unfolded protein likely precipitated and did not reach the detector. Both DMSO and methanol addition prompted the appearance of a new set of peaks, with masses corresponding to CCT dimers (Fig. 4c). We used subunit mass measurements (Supplementary Data 2) to assign stoichiometries to the dimers. We found that the majority of dimers contained CCT5, with the most abundant subunit pair in either solvent being a CCT5-CCT5 homodimer (Fig. 4d; Supplementary Data 4). In total, whereas CCT5 comprised 31% and 42% of the total monomer signal after DMSO and methanol addition, respectively, 58% and 70% of the dimer signal arose from up to seven different dimer species that contained CCT5 (Fig. 4d; Supplementary Data 4). While the overall dimer profiles after DMSO and methanol addition were similar, more unique dimers overall were detected in the DMSO condition compared to methanol, with CCT8-CCT8 constituting roughly 15% of all dimer signal in this condition only (Fig. 4d). Detecting additional complexes in the presence of DMSO is not unexpected because DMSO, as a subcharging agent, can preserve particularly labile protein–protein interactions during ionization^23^.

We next categorized all potential dimers as canonical (i.e. contacts present in the original native TRiC sample) or non-canonical (i.e. contacts between subunits that do not interact in the original complex). An analysis of the surface areas and physicochemical properties of the various interfaces in the hetero-oligomer (Methods) led us to classify subunits sharing intra-ring contacts between adjacent domains as canonical dimers; less extensive interfaces such as inter-ring and cross-ring contacts were found to be much weaker and were not included﻿. Canonical dimers could represent disassembly intermediates of the intact complex whose contacts survive complex disruption by solvent or could be formed by specific interactions between dissociated monomers. A random dimer pairing between the eight subunits has a roughly 1 in 5 chance of being canonical. Surprisingly, over two thirds of all unique dimers observed after DMSO or methanol disruption involved non-canonical contacts; by abundance, almost 90% of all dimer signal arose from non-canonical pairings, many of which included CCT5 (Fig. 4d; Supplementary Data 4). Of note, cross-linking MS (Supplementary Data 1) and previous structural analyses^10^ indicate that the subunits in the original hTRiC sample are arranged in the canonical subunit arrangement. Thus, the non-canonical dimers observed in denaturing conditions likely originate by re-association of monomers, rather than disassembly intermediates of the complex. Because dissociation into monomers began prior to addition of organic solvent, as shown in Fig. 2, we revisited native MS data without DMSO or methanol seeking evidence of dimer formation even at 100-fold lower monomer concentrations. Indeed, dimer peaks clearly assignable to CCT5-CCT5 were found to be present before complete disruption of the complex; this signal was even lower when the complex was stabilized by ATP (Supplementary Fig. 3). Altogether, it follows that most if not all of the dimers obtained in dissociation experiments necessarily formed by re-association after passing through a transient monomeric state.

## Discussion

Various mass spectrometry-based approaches have been applied in this study to characterize recombinant human TRiC. The chaperonin adopts the correctly assembled 16-membered ring arrangement and is a functional chaperone for actin folding. Two alternative purification pipelines yield ~ 95% purity^10^, co-purifying with trace amounts of functionally related proteins from the expression system, *T. ni*.. These include insect CCT; chaperones Hsp70 and Hsp90 and endogenous substrates, most abundantly tubulin. It is possible that small amounts of assembled *T. ni* TRiC/CCT co-purify through the steps in our protocol; alternatively, we cannot discount a small amount of co-assembly between insect CCTs and human TRiC, based on sequence conservation of TRiC between these species (Supplementary Fig. 4) and across eukaryotes generally^24^.

Proteomic analysis indicated that hTRiC can contain either α- or β-tubulin remaining bound at the end of a preparation. The occupancy of this substrate varies from less than 10% to approximately half of all double-ring chambers (Supplementary Data 3). Inclusion of an ATP wash prior to affinity purification can greatly reduce the amount of bound substrate^25^. Addition of tubulin folding cofactors or tubulin heterodimers and GTP to promote release and polymerization may further reduce tubulin co-purification^26^. Actin, though also an obligate substrate of TRiC, was detected only at trace levels compared to tubulin in one replicate analysing the entire sample and was not found when the complex was excised from native gels. Hsp70 co-purification is likely related to its functional cooperation with TRiC in cotranslational folding^27,28^. A direct interaction between Hsp70 and subunit CCT2 has been observed^29^. The mechanistic underpinnings of potential Hsp90-TRiC interaction are less understood, though this chaperone, like Hsp70, has also long been associated with TRiC in various model systems^30,31^. Several ribosomal proteins were also detected at low levels that did not meet all thresholds for significance. While physical interactions between CCT subunits and the ribosome have been reported^17^, they did not appear to persist in our data, as ribosomal subunits were only detected in solution and not as species co-migrating with hTRiC in Native PAGE. Overall, the inclusion of trace amounts of substrates, homologs, and additional proteostasis factors in the purified samples is important to be aware of but not of major concern. At approximately 70% sequence identity and 90% similarity (Supplementary Fig. 4), the 2–4% abundance of highly conserved expression system subunits will not be a hindrance in the majority of conceivable applications for recombinant hTRiC. Inclusion of additional purification steps could be desirable when > 99% purity and absence of tubulin is required.

Using a combination of intact mass measurements of isolated CCT subunits and top-down mass spectrometry, we have deduced the N-terminally modified state(s) of all eight hTRiC subunits in the recombinant system (Fig. 3f). This approach bypasses the need for chemical enrichment strategies normally employed to identify PTMs; another recent example is its application to infer N-terminal processing of the proteasome^32^. The observed hTRiC processing is consistent with the expected specificity of N-terminal modification enzymes, methionine aminopeptidases (MAPs) and N-terminal acetyltransferases (NATs)^33,34^. The initiator methionine was found to be absent when the subsequent residue contained a small side chain (alanine, glycine, or proline) and present when the next residue was bulky or charged (glutamate). For each subunit that encodes methionines at both the first and second residues, CCT3 and CCT7, we observed masses consistent with two states, where neither or both methionines were cleaved. Masses were consistent with N-terminal acetylation wherever a primary amine was exposed, with the exception of the proportion of CCT3 beginning with glycine, a residue that is rarely acetylated^35^.

To assess whether the recombinant proteoforms reflect in vivo processing, we compared these modifications to several prior proteomic studies describing the N-terminal states of TRiC subunits (Supplementary Data 2)^36–38^. A compilation of N-terminal modifications for all TRiC/CCT subunits across eukaryotic organisms showed broad agreement between the N-terminal proteoforms in recombinant hTRiC and its subunits in vivo*,* even though not all datasets agreed with each other or detected all modifications. At least one identically modified form of each of the eight subunits was previously identified in datasets obtained from human cells, and all modifications profiled in a study of mouse tissue were also consistent with our results. Not all studies surveyed identified both states of CCT3 and CCT7 observed here: each was more likely to be acetylated without methionine excision than to lose two methionines. In addition, one prior study identified CCT4 as having lost both methionine and its second residue, proline, whereas we observe only methionine loss^36^. Other studies reported CCT4 modification consistent with ours^37,38^. These partial deviations may reflect cell type specific mechanisms of MAPs or NATs. Alternatively, the proportion of proteins modified is more likely to differ than the rules by which they are modified^33,34^. Kinetic competition between MAPs and NATs may also play a role, with acetylation of initiator methionines preventing their removal by MAPs^39,40^. Overall, we identify physiologically relevant N-terminal processing of recombinant hTRiC.

These results may be used to infer the likely state of the N-terminus in other organisms based on subunit sequences. For example, yeast CCT1, CCT2, CCT4, CCT5, CCT6, and CCT8 encode serine or alanine in the second position and would be expected to undergo methionine loss and acetylation; whereas yeast CCT3 and CCT7, with glutamine and asparagine in position two, respectively, should only be acetylated. Three studies profiling the yeast N-terminome were found to be in full agreement with these predictions (Supplementary Data 2)^37,41,42^.

Whether and how N-terminal processing is important for TRiC assembly or function remains to be determined. The N-termini are located within the folding chamber, and have been proposed to participate in an allosteric network by contacting helices of neighboring subunits^5^. Truncating the N-terminus of CCT4 or CCT7 in yeast results in growth defects^5^. Acetylation can also act as a degradation signal via the N-end rule (or N-degron) pathway, an arm of the ubiquitin–proteasome system^43,44^. Acetylation might help the cell to dispose of subunits that do not assemble quickly enough to bury their N-termini, an established mechanism for quality control of multiprotein complexes^43^. Suggestive of this, CCT4, the only completely non-acetylated subunit in our system, has a longer half-life in MCF-7 cells than any other CCT protein except CCT1^45^. The same study also found a much stronger effect of proteasome inhibition compared to autophagy inhibition on CCT half-lives, consistent with earlier data implicating the proteasome in CCT degradation^45﻿,46^. It will be interesting to investigate in greater detail whether differential N-terminal modification of CCT subunits affects their turnover.

Toward dissecting the mechanism of TRiC assembly, we used high resolution native mass spectrometry to detect subcomplexes in the presence of solvent that disrupts the intact 16-mer complex. This led to the identification of 20 different dimer pairs forming between CCT subunits. Surprisingly, most of the dimers we detected comprised subunits engaging in non-canonical contacts, containing interfaces that do not exist in correctly assembled TRiC, suggesting they re-assemble after dissociation (Fig. 4e). The dimers formed were similar for two dissociation conditions, and the most abundant dimer, CCT5-CCT5, was also present in no-solvent conditions, indicating that dimer formation depends at least in part on monomer concentration (Supplementary Fig. 3). CCT5 contributed up to 70% of all dimer signal and formed dimers with all other subunits except CCT8. Relative to other subunits, CCT5 was also abundant as a monomer in native MS and exhibited high recovery following RP-HPLC separation of the complex, which coincided with elution of partially unfolded dimers (Fig. 1c–d). These findings altogether indicate that CCT5 is more stable than other subunits under chemical destabilization, and more likely to self- and co- assemble.

Our findings resonate with previous studies showing that, when expressed in *E. coli,* some TRiC/CCT subunits form non-canonical homo- and hetero-oligomeric assemblies. For instance, CCT5 can homo-oligomerize when expressed in *E. coli,* assembling into a full 16-mer double ring complex^47,48^, and recombinant CCT5 can bind paralogs promiscuously when overexpressed in pairs in *E. coli* ^17^. Thus, CCT5 can interact with many other CCT subunits even in cells. Recombinant CCT4, CCT1, CCT2, and CCT6 can also homo-oligomerize in the absence of other subunits^48,49^. While we did detect CCT4-CCT4 and CCT2-CCT2 dimers after RP-HPLC, these dimers were not especially abundant in our native MS dissociation analyses. We detected little to no homodimer signal in our data for CCT1, CCT2, and CCT6, indicating that for these, interactions with other subunits are more favorable than homotypic interactions. In addition, these subunits were not as stable as CCT5 under the experimental conditions. The greater stability of CCT5 and its ability to interact with multiple CCT subunits, including itself, suggests this subunit may play a key role during TRiC assembly.

While the possible relevance of these non-canonical dimers, formed here after in vitro dissociation of the TRiC complex, remains unclear, their formation suggests that CCT subunits have plastic inter-subunit interaction interfaces. This raises the question of what mechanisms lead to the formation of a unique correct arrangement in vivo. It should be informative to investigate the sequence determinants of these subunits’ canonical and non-canonical interfaces and how a subunit’s capacity for homo- or hetero-multimerization relates to the assembly process. Our experiments indicate that the information leading to the correct subunit arrangement in TRiC is not directed by the intrinsic affinity of subunit interfaces for each other, consistent with prior failed attempts to assemble the complex from its components ex vivo^16^.

In principle, off-pathway dimers may form during TRiC assembly and could trigger pairwise recognition by degradation factors. Alternatively, non-canonical CCT species could form at low levels and have “moonlighting” functions, as observed for other molecular chaperones^50^. One early study estimated that 5 to 15% of the CCT proteins in mouse P19 cells are not assembled in TRiC but instead are small species consistent with dimers or trimers, with subunit pairs exhibiting partly non-overlapping subcellular localizations evidencing independent functions^51^. Another early study also identified unassembled “micro-complexes” and, reasonably assuming that they originated directly from the holocomplex, used them to assign a ring arrangement that was later found to be incorrect^7^. Since these early reports, in vivo CCT non-canonical associations and functions have received little attention. Cellular phenotypes unique to over-expression of a single subunit without concurrent TRiC up-regulation have been reported for CCT4 and CCT5^52,53^. Our study is the first to offer quantitative insight into which CCT-CCT contacts can form when all subunits are present at low concentrations. Whether these contacts reflect micro-complexes previously observed in vivo or only form during in vitro dissociation will be subject to future work.

Another explanation for non-canonical subunit interface compatibilities is that they are remnants of the evolution of the TRiC hetero-oligomer. All CCT subunits inherited an oligomeric interface from an ancient, single-copy CCT gene that duplicated several times deep in the eukaryotic lineage^54^. In many paralogs that interface was likely only partly lost as their specific positions in the ring were entrenched, as has been demonstrated for other multimeric complexes with ring topologies^55,56^. Our results could be explained if the residual affinity between paralogs that are not adjacent in TRiC reflects incomplete loss of ancient contacts ^7,17,47–49^﻿. Whether they signify moonlighting functions or byproducts of evolution, non-canonical CCT pair contacts must be prevented or displaced during TRiC assembly by kinetic or competitive barriers (Fig. 4e).

**Figure 5:**
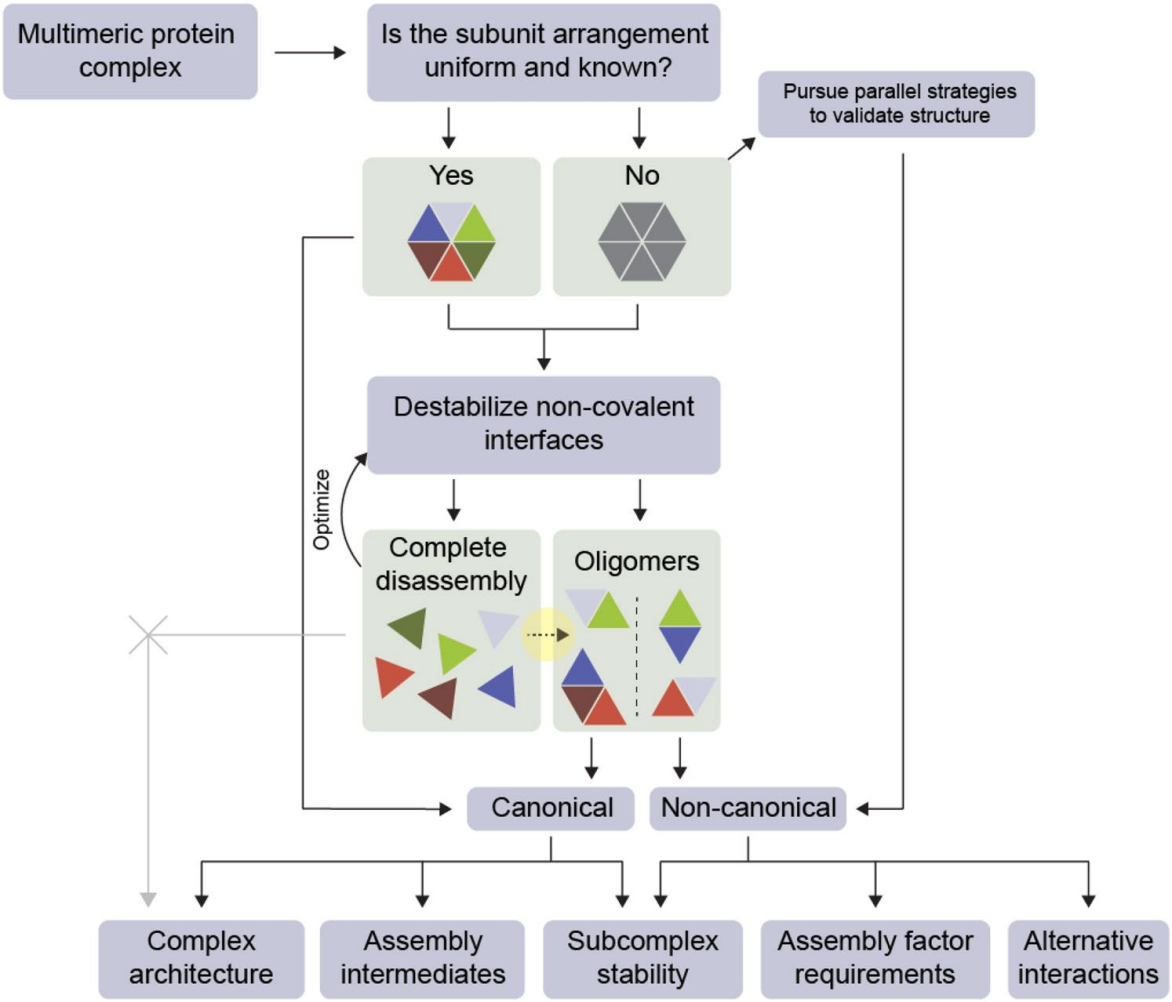
Updated conceptual workflow for solution dissociation coupled to native mass spectrometry. The highlighted arrow from *Complete disassembly* to *Oligomers* represents the novel pathway observed in this study, which can occur if, for example, subunits possess moonlighting functions with alternative partners or are paralogous and retain non-canonical interface compatibility.﻿ Assignment of contacts as canonical or non-canonical ensures that native MS is appropriately applied in integrative structural biology pipelines.

Solution dissociation coupled to native MS has been established for studying arrangements, assembly and disassembly pathways, and evolutionary histories as part of an integrated approach to the structural analysis of protein complexes^18–22^. In a departure from most prior applications, our aim was not to discern which components of a multimer are adjacent to each other, as the subunit arrangement of TRiC is known, but rather to identify (dis)assembly intermediates. The extent of non-canonical re-associations that we measure suggest that when using this approach as a tool for topological studies of protein complexes, care should be taken to discern whether contacts identified by native MS are canonical or non-canonical. Because non-canonical interfaces are distinct from artefactual interfaces in that they represent contacts that can affirmatively occur in vivo unless they are disfavored, the benefit of this additional step is that information can be gleaned beyond the binary existence or non-existence of particular protein–protein contacts (Fig. 5). Due to the evolutionary implications discussed above, we suspect these recommendations are particularly salient where paralogous subunits are involved. The results also suggest a way to harness the high mass resolution afforded by native MS to diagnose and further investigate facilitated assembly along multimeric pathways. For example, designating the subcomplex profile of TRiC under a particular destabilizing condition as a sort of fingerprint could serve as a baseline for the re-addition of putative assembly factors in search of specific effects on intermediates that would be difficult to capture by alternative techniques.

Any intermediates in the course of forming an ordered hetero-oligomer are likely to be energetically favored only on the timescale of assembly to facilitate efficient formation of the final, functional products^18,57^. Thus, the apparent differences in stability that we observed—CCT5 and CCT8 among the most stable, CCT1 and CCT7 the least—may reflect these subunits’ roles in the assembly process. Examining TRiC turnover and subcomplex formation using different combinations of CCT proteins and co-factors in vivo and in vitro will shed more light on its highly regulated assembly.

## Methods

### Molecular cloning

Cloning of the baculovirus insect vector co-expressing all human CCT subunits was adapted from^58^. First, 4 CCT subunits from two hemispheres were cloned into vector pBIG1a or pBIG1b. Gene fragments encoding CCT1, CCT8, CCT6 and CCT3 were PCR-amplified (primers, Supplementary Table 1) from pFasbacDual parental vectors (Supplementary Table 2) and gel purified. pBIG1a was linearized using restriction enzyme SwaI. Gibson assembly reactions were carried out to generate pYC31 (pBIG1a-CCT1-CBP, CCT8, CCT6, CCT3) or pYC32 (pBIG1a-CCT1, CCT8, CCT6, CCT3)^59^. CCT2, CCT4, CCT7 and CCT5 were similarly cloned into pBIG1b to produce pYC33 (pBIG1b-CCT2, CCT4, CCT7, CCT5) or pYC34 (pBIG1b-CCT2, CCT4, CCT7-6 × His tag, CCT5). To assemble all CCT subunits into a single vector, pYC31 and pYC33 were digested using restriction enzyme PmeI and the gene fragments containing CCT coding sequences were excised and purified. Vector pBIG2ab was also linearized by PmeI and mixed with CCT gene fragments to produce pYC35 (pBIG2ab-TRiC, CCT1-CBP) by Gibson assembly. CCT gene fragments excised from pYC32 and pYC34 were assembled into pBIG2ab vector to generate pYC36 (pBIG2ab-TRiC, CCT7-6 × His).

### Protein expression and purification

Complexes were expressed as described previously^10^ using the Bac-to-Bac baculovirus expression system (Invitrogen). 2 L of cells infected with pYC36 P3 virus were harvested after 48 h and resuspended in 100 ml lysis buffer, consisting of 20 mM HEPES pH 7.4, 50 mM NaCl, 5 mM MgCl2, 10% glycerol, 1 mM DTT, PMSF, complete protease inhibitor cocktail (Roche), and DNAseI (Sigma-Aldrich). Cells were lysed by sonication, and lysate was clarified by centrifugation at 20,000 × g for 20 min. hTRiC containing 6 × His-tagged CCT7 was purified by ammonium sulfate precipitation, followed by overnight dialysis in MQA buffer (20 mM HEPES pH 7.4, 50 mM NaCl, 5 mM MgCl2, 10% glycerol, 1 mM DTT, 0.1 mM EDTA pH 8.0). Lysate was diluted in 1X MQA buffer and loaded onto a FFQ column, washed in 5% MQB buffer (20 mM HEPES pH 7.4, 1 M NaCl, 5 mM MgCl2, 10% glycerol, 1 mM DTT, 0.1 mM EDTA pH 8.0) and eluted in 75% MQB. Eluate was passed through a heparin column, washed with 30% MQB and eluted in a 400 ml gradient from 30 to 80% MQB. Fractions were analyzed by 10% SDS-PAGE, and those containing hTRiC were concentrated to 2 ml by centrifugation in a centricon plus-700, 100 kDa MWCO (Millipore). Concentrated hTRiC was purified by SEC (Superdex200 26/60, GE Healthcare) in MQA buffer. hTRiC-containing fractions were identified by SDS-PAGE, concentrated to 300 μl, aliquoted and snap frozen.

To express hTRiC (CCT1-CBP), 1 L of Hi5 cells were infected with pYC35 P3 virus for 55 h, harvested, and resuspended in 100 ml MQA buffer with 2 mM CaCl_2_. Cells were lysed in an Emulsiflex (Avestin), clarified by centrifugation at 50,000 × g for 1 h and purified using calmodulin affinity resin (Agilent Technologies). The column was washed in MQA with 150 mM NaCl, 2 mM CaCl_2_ and 0.05% NP-40 and complex was eluted in MQA with 150 mM NaCl and 2 mM EGTA. The sample was further purified by anion exchange chromatography using a monoQ 10/100 column equilibrated with MQA buffer in a 150 ml gradient to 100% MQB. Eluted hTRiC was assessed by 10% SDS-PAGE, and pure fractions were concentrated in an Amicon Ultra-4 centrifugal filter (Millipore) and purified by SEC using a Superose 6 26/60 (GE Healthcare) equilibrated in MQA buffer.

For hTRiC lacking CCT2 and CCT4 (which contained 6 × His-tagged CCT7), 1 L of cells was infected with three P3 viruses (pDG463, pDG445 and pDG446) each encoding a CCT pair, harvested after 72 h, and lysed in buffer consisting of 50 mM HEPES pH 7.4, 100 mM NaCl, 4 mM imidazole, 0.05% NP-40, 10% glycerol, PMSF, complete protease inhibitor cocktail (Roche), and benzonase 5 U ml^−1^ (Sigma-Aldrich). Cells were lysed in an Emulsiflex (Avestin), lysate clarified at 50,000 × *g* for 1 h and purified by Ni Sepharose HP (GE Healthcare). Eluate was further purified by SEC (Superdex200 26/60, GE Healthcare) equilibrated in MQA buffer. Fractions were identified by SDS-PAGE, concentrated to 300 μl in an Amicon Ultra-4 centrifugal filter (Millipore), aliquoted and snap frozen.

### Structural and functional validation

Clear native gel electrophoresis was performed using a 4–16% Bis–Tris (pH 7.4) gel system from Invitrogen per manufacturer instructions. The gel was stained with GelCode blue stain reagent (Thermo Fisher Scientific). The proteinase K (PK) conformational cycling and radiolabeled actin folding assays were performed as described previously^10^. For negative stain electron microscopy, hTRiC was diluted 10 times to 0.05 mg ml^−1^ in 20 mM HEPES, 50 mM NaCl, 5 mM MgCl_2_, 1 mM EDTA, 1 mM DTT. 5 μl sample was placed for 1 min on a formvar/carbon coated 200 holey mesh Ultra Thin copper grid (FCF200-Cu-UA, Electron Microscopy Sciences). The grid was blotted on filter paper, floated over stain for 1 min in 1% uranyl formate made fresh from powder (Electron Microscopy Sciences), and air dried. Grids were imaged using a JEOL JEM1400 transmission electron microscope.

### Reverse phase chromatography

Individual hTRiC subunits were separated by RP-HPLC (column 214TP54, Vydac). Each complex was loaded (200 μg), washed with 10 ml of 45% acetonitrile, and eluted in a gradient of 45–70% acetonitrile/0.1% TFA over 40 ml. Individual fractions were collected and concentrated to 20 μl in 500 μl Amicon ultra centrifugal filters (Millipore). Samples were then divided for (i) analysis by 10% SDS-PAGE, stained with coomassie blue; (ii) intact MS; or (iii) Western blotting, via transfer to a nitrocellulose membrane and immunoblotting against each subunit for band identification. Santa Cruz antibodies were used for: CCT1 (sc-374088), CCT4 (sc-137092), CCT6 (sc-514466), CCT7 (sc-271951), and CCT8 (sc-377261); Abcam antibodies were used for: CCT2 (ab92746), CCT3 (ab106932), CCT5 (ab129016).

### Denaturing intact mass spectrometry

Fractions eluted from the reverse phase column were stored on ice and analyzed within 48 h in the acetonitrile/TFA elution buffer. Concentrations were estimated based on UV absorbance to be between 0.1 and 0.6 μM. Attempts to exchange the buffer or dry the sample and resuspend in a new buffer consistently resulted in loss of the sample, therefore it was necessary to load enough intact hTRiC onto the column to recover sufficient quantities of separated subunits for direct analysis. Samples were loaded into borosilicate emitters (ThermoFisher Scientific ES380) and injected by nanoelectrospray into a QExactive Plus EMR (Extended Mass Range) orbitrap mass spectrometer (ThermoFisher Scientific). Capillary voltage was 1.3 kV, source temperature 250 °C, and S-lens RF (radio frequency) level 100. The mass range was set to 900–10,000 m/z, operating the quadrupole in RF-only mode. Ion injection time was 50 ms. Resolution was 17,500 at 200 m/z for a transient time of 64 ms. Scans were acquired in groupings of 10 microscans without averaging. In-source activation was applied at 70 V to improve signal through desolvation. The higher-energy collisional dissociation (HCD) cell activation energy was 30 V. Pressure in the HCD cell (nitrogen) was kept between 4–6 × 10^−10^ mbar. Optics were tuned for transmission as needed, deviating little from the following settings: source DC offset 25 V; injection flatapole 14 V; inter flatapole lens 10 V; bent flatapole 7 V; and C-trap entrance lens 2 kV. Calibration was performed under the same settings using clusters of CsI over the relevant mass range.

To measure intact masses via LC–MS (Supplementary Fig. 2), 1 μg of hTRiC was passed over a C18 column (Acclaim PepMap 100, 75 μm × 15 cm, ThermoFisher Scientific) by a Dionex UltiMate 3000 RSLC nano system, then continually injected into a hybrid LTQ-Orbitrap XL mass spectrometer (ThermoFisher Scientific) operating in positive ion mode. A blank was run prior to hTRiC injection. The sample was equilibrated in buffer A (0.1% v/v formic acid) and separated by a linear gradient of 1 to 99% buffer B (5:45:50 v/v H_2_O:ACN:IPA with 0.1% v/v formic acid) at a flow rate of 300 nl min^−1^. Mass spectra were acquired by orbitrap scanning with a resolution of 60,000 from 335 to 2000 m/z.

Raw data from offline MS (Fig. 1) were summed in the Qual Browser of XCalibur v4.1.31.9 (ThermoFisher Scientific) and exported to UniDec v3.1.0^60^ for visualization. A curved baseline subtraction was applied as needed. Mass spectra for CCT1 and CCT7 were Gaussian smoothed with width 3. Masses reported in Supplementary Data 2 (monomers) and Supplementary Data 4 (dimers) were extracted manually by minimizing error over different charge state assignments using the Mass and Charge State Evaluation and Determination tool (MaCSED v0.3) available online at benesch.chem.ox.ac.uk/resources.html. Errors in denatured dimer masses were determined using monomer masses measured in the same spectrum and assuming disulfide formation (-2 Da). Dimer charge series were also fit to monomer sized species, resulting in errors roughly 10 × higher and monomer masses that did not match CCTs; thus, the possibility that these peaks represented monomers was excluded. Raw data from online LC–MS (Supplementary Fig. 2) were summed in XCalibur.

### Native mass spectrometry

Samples were buffer exchanged into ammonium acetate (ranging from 250 mM to 1 M pH 7.5, −/+ 500 µM ATP where indicated in the text) using a minimum of five resuspensions in Amicon centrifugal filters with 50 kDa molecular weight cutoff (Millipore), then kept on ice and analyzed within 48 h. This method of buffer exchange was found to remove salt and glycerol more effectively than alternatives based on removal of monomer adducts in the data. For dissociation experiments, LC–MS-grade DMSO or methanol were added to hTRiC to a final concentration of 25% (v/v) and analyzed within 30 min. Gold-plated borosilicate capillaries prepared in-house were used to inject samples by nanoelectrospray into a Q-Exactive Plus UHMR (Ultra-High Mass Range) orbitrap mass spectrometer (ThermoFisher Scientific)^11^. Capillary voltage was 1.2–1.4 kV in positive ion mode. Source temperature was 200 °C and S-lens RF was 200%. Nitrogen was used in the HCD cell at pressure 1.3–1.5 × 10^−9^ mbar. In-source trapping and HCD voltages were tuned to maximize signal intensity while avoiding fragmentation for each sample, 50 V and 40 V, respectively. Optics were tuned to transmit and optimally resolve either megadalton complexes or lower mass monomers and dimers by adjusting the injection flatapole, inter-flatapole lens, bent flatapole, and transfer multipole voltages while leaving the C-trap entrance constant at 5 kV. Resolving power was 17,500 at m/z 200 for a transient time of 64 ms and the noise threshold was 3. The automatic gain control (AGC) target was 1 × 10^6^. Once a stable spray was achieved, transients were combined in groupings of 30 microscans.

Raw data were summed in XCalibur for visualization. Peaks belonging to the same charge state distributions were identified in UniDec and the monomer masses in Supplementary Data 2 were then extracted manually using MaCSED v0.3. These were compared to theoretical average monomer masses generated using ProtPI (protpi.ch). Acquisition times included in data summations were normalized before directly comparing monomer signal intensities (Fig. 4a).

### Dimer categorization, assignments, and quantification

To assign dimers, mass spectra were first deconvolved in UniDec using the parameters listed in Supplementary Data 4 to generate a list of species present. This list was curated manually by removing a minority of false identifications whose charge state distributions were not Gaussian. The resulting list was compared with dimer masses calculated using experimental monomer masses from the same instrument on the same day, and considered to match if the assignment was within 12 Daltons (0.01% error) and unambiguous with respect to all other theoretical dimers containing hCCT and/or tCCT subunits (tCCT masses were predicted based on sequences used in proteomic searches with N-terminal processing analogous to hCCT). Relative intensities of the deconvolved dimers were recorded from UniDec. In the case of DMSO addition, contributions were subtracted from single trailing peaks that could be assigned to overlap with charge-reduced monomers in four separate homodimer assignments. We attempted to assign the minority of unknown species in the dimer regions to contacts involving one or two tCCT subunits, but were unable to assign any of these to *T. ni* CCT proteins with certainty.

To designate canonicality, the PDBePISA server was used to analyze the chemical properties of subunit interfaces^61^. To maximize interface contacts, we submitted the structure of yeast TRiC in the closed conformation (PDB 4V94; equivalent not available for human TRiC). Each lateral inter-ring interface was found to contribute a predicted − 30.5 kcal mol^−1^ on average to the free energy of complex formation, whereas an aligned intra-ring contact contributes only approximately − 2.4 kcal mol^−1^. Moreover, the PDBePISA analysis revealed a third viable interface type, where intra-ring contacts between N-termini within the chamber contact diagonally offset subunits in the opposite ring. Each of these ‘interfaces’ is predicted to contribute − 2.6 kcal mol^−1^, essentially equivalent to aligned interfacial areas. If the dimers detected by native mass spectrometry existed in multiple conformations reflecting the retention of different types of interfaces, we would expect a range of charge state distributions that reflect different solvent accessible surface areas. This was not observed; instead, the data pointed to a common dimer conformation. Thus, we designated only theoretical dimers containing inter- and not intra-ring subunit interfaces as canonical. Canonical fractions by assignment were calculated using the list of all dimers. Fractional abundances of each subunit were calculated using relative dimer intensities, divided by two in the case of heterodimers.

### Top-down mass spectrometry

A native mass spectrum of hTRiC lacking CCT2 and CCT4 was first collected on the QExactive UHMR as described, with optics tuned to transmit monomers. Next, the quadrupole was switched from RF-only to isolation window was set to 8 Daltons centered on a monomer peak; resolving power was raised to 140,000 to allow longer transient times; UHV pressure (nitrogen) was lowered to 3 × 10^−10^ mbar; the scan range was changed to 500–10,000 m/z; and the AGC target was lowered to 5 × 10^5^. Upon confirming transmission of the peak of interest, the HCD energy was raised to 150 V to provoke fragmentation and scans were summed for an acquisition time of 8 min and 800 microscans. This was repeated for three CCT5 charge states.

Raw data were summed in XCalibur and deisotoped using the Xtract function. The deisotoped mass spectra were exported as peak lists, and all peaks with raw intensity less than 50 (~ 3% of the maximum intensity) were discarded. The curated lists were individually input into ProSight Lite v1.4^62^ and used to search against each hCCT sequence individually, with variable N-terminal processing states and stringency of 10 ppm. Reported fragment matches in Fig. 3 correspond to the N-terminal states in Supplementary Data 2.

### Proteomics

100 μg hTRiC (CCT1-CCBP or CCT6-GFP) was denatured in 8 M urea, reduced in 5 mM DTT (Sigma-Aldrich), and incubated at 60 °C for 30 min. Samples were alkylated in 15 mM iodoacetamide solution and incubated in the dark at room temperature for 45 min. Digestion was performed overnight in 100 ng trypsin (sequence grade, Promega) and peptides were purified and concentrated using Zip-Tip C18 Cartridge columns (Millipore) then resuspended in 10 μl 0.1% formic acid for MS analysis. The hTRiC complex band excised from clear native-PAGE was digested in-gel. Proteins in the gel band were de-stained in 25 mM NH_4_HCO_3_ in 50% acetonitrile and reduced in 10 mM DTT at 56 °C for 1 h. The sample was alkylated with 27.5 mM iodoacetamide solution in the dark at room temperature for 45 min, dehydrated in 25 mM NH_4_HCO_3_ in 50% acetonitrile, and dried completely in a speed-vac. The gel was then covered in 3X its volume with 12.5 ng ul^−1^ trypsin in 25 mM NH_4_HCO_3_, rehydrated on ice for 10 min, and incubated overnight at 37 °C. Peptides were extracted in 50% acetonitrile / 5% formic acid, and the sample was placed in a speed vac until the volume reduced to 10 μl. Peptides were cleaned using a Zip-Tip C18 cartridge column (Millipore) then resuspended in 10 μl 0.1% formic acid.

The LC–MS/MS analysis was performed by a Velos Pro Elite Orbitrap Mass Spectrometer (Thermo Fisher Scientific) coupled with a NanoAcquity UPLC system (Waters) through an Easy-Spray PepMap column (75 μm x 15 cm, Thermo Fisher Scientific). During the LC separation, 0.1% formic acid in water was used as the mobile phase A while 0.1% formic acid in acetonitrile was used as the mobile phase B. Phase A and phase B were mixed at the point of entering the Easy-Spray PepMap column. Following the initial column equilibration in 98% A / 2% B over 20 min, the concentration of the phase B was linearly increased from 2 to 30% at a flow rate of 300 nl min^−1^ over 27 min. Then the phase B concentration was increased linearly from 30 to 50% sequentially over two minutes. The column was then re-equilibrated in 98% A / 2% B over 11 min.

The MS/MS data was acquired in a “top six” data-dependent sequence. After a survey scan, the six most intense precursor ions were selected for subsequent fragmentation using the higher-energy collisional dissociation (HCD) technique with normalized collision energy of 27.5 V. Both precursor and fragment ions were analyzed in the Fourier Transform (FT) mode in the Orbitrap at mass resolution of 60,000 and 15,000, respectively.

Data were processed using MaxQuant v1.6.14^63^. Searches were performed against the *Trichoplusia ni* proteome (downloaded from tnibase.org); the human CCT proteins expressed; and a list of common contaminants included in MaxQuant. Several *T. ni* CCT protein sequences in the tnibase.org database were found to be incomplete, so all were replaced with the eight results of a NCBI protein BLAST search against the *T. ni* proteome using human CCT5 as query. NCBI accession numbers of the sequences used are provided in Supplementary Data 3. The minimum peptide length was 7 residues. Intensity-based absolute quantification (iBAQ) was enabled^64^. The minimum number of unique peptides was set to 1. Trypsin was set as the enzyme with a maximum of 2 missed cleavages. Carbamidomethylation of cysteines was a fixed modification; oxidation of methionine and acetylation at the N-terminus were variable modifications. The first peptide search tolerance was 20 ppm and the main peptide search tolerance was 4.5 ppm. The false discovery rate (FDR) for peptides and proteins was 1%. The output was analyzed in Perseus v1.6.5^65^. Matches only identified by site; reverse matches; and contaminants were removed. The list was then curated to contain only proteins with at least 2 unique peptides and scores greater than 1. Finally, relative iBAQ (riBAQ) values were calculated as fractions of the summed iBAQ intensity^64^. Solution data is presented as the average of two replicates collected on different dates, where all components met thresholding criteria in both samples. The maximum fraction of hTRiC associated with tubulin was calculated using the summed estimated abundance of all detected tubulin isoforms and all hTRiC subunits, assuming that a 16-subunit chamber with occupancy 1 tubulin would manifest as 1/17th tubulin by riBAQ.

### Cross-linking mass spectrometry

Samples were cross-linked using the zero-length cross-linking reagent DMTMM (4-(4,6-Dimethoxy-1,3,5-triazin-2-yl)-4-methylmorpholinium chloride) and analyzed as described previously^66^. Briefly, the cross-linked sample (approximately 50 µg protein) was digested with endoproteinase Lys-C (2.5 h, 1:100 enzyme-to-substrate ratio) and trypsin (overnight, 1:50). The digested sample was purified by solid-phase extraction and fractionated using peptide-level size-exclusion chromatography (SEC; Superdex Peptide, GE). Three fractions collected from the SEC experiment were analyzed in duplicate by LC–MS/MS on an Orbitrap Elite mass spectrometer (ThermoFisher Scientific) in data-dependent acquisition mode with 60 min gradients. MS/MS data was searched using xQuest^67^ against a database containing the TRiC subunits and contaminant proteins, and identifications were filtered at a false discovery rate of 5% at the peptide pair level as determined by xProphet. Distances were extracted using PDB (Protein Data Bank) entry 6NRA as a structural model.

## Supplementary Information

Supplementary Information 1.

Supplementary Information 2.

Supplementary Information 3.

Supplementary Information 4.

Supplementary Information 5.

Supplementary Information 6.

Supplementary Information 7.

## Data Availability

Filtered results of proteomic analyses are provided in Supplementary Data files 1 and 3. Quantification of native mass spectrometry results is provided in Supplementary Data 2 and 4. Raw source data is available from the corresponding author upon reasonable request.
