# Supplementary material for: Native mass spectrometry analyses of chaperonin complex TRiC/CCT reveal subunit N-terminal processing and re-association patterns
Source: Sci Rep. 2021 Jun 22;11:13084. doi: 10.1038/s41598-021-91086-6 (PMC8219831; doi:10.1038/s41598-021-91086-6)
Supplement: Supplementary file 1 — Supplementary Information 1. [file 41598_2021_91086_MOESM1_ESM.docx]

Supplementary Material

Native mass spectrometry analyses of chaperonin complex TRiC/CCT reveal subunit N-terminal processing and re-association patterns

Miranda P. Collier^a^, Karen Betancourt Moreira^a^, Kathy H. Li^b^, Yu-Chan Chen^a^, Daniel Itzhak^c^, Rahul Samant^a^, Alexander Leitner^d^, Alma Burlingame^b^, Judith Frydman^a^*

*Correspondence to: jfrydman@stanford.edu, +1 650 725 7833

*^a^ Department of Biology, Stanford University, Stanford, CA, US*

*^b^ Department of Chemistry, University of California San Francisco, San Francisco, CA, US*

*^c^ Chan-Zuckerberg BioHub, San Francisco, CA, US*

*^d^ Department of Biology, Institute of Molecular Systems Biology, Zurich, Switzerland*

**Supplementary Data Files**

**Supplementary Data 1.** Cross-linking mass spectrometry analysis of hTRiC.

**Supplementary Data 2.** Measured masses and N-terminal modifications of recombinant hTRiC subunits.

**Supplementary Data 3.** Proteomics of solution- and NativePAGE-derived hTRiC.

**Supplementary Data 4.** Masses and quantification of CCT dimers detected by native and denatured intact mass spectrometry.

**Supplementary Information** contains Supplementary Figures 1-5 and Tables 1-2:

**Supplementary Figure 1.** Structural and functional validation of recombinant hTRiC.

**Supplementary Figure 2.** Intact LC-MS of hTRiC allows orthogonal mass measurement.

**Supplementary Figure 3.** Sequence alignments of *H. sapiens* and *T. ni* CCT subunits.

**Supplementary Figure 4.** Formation of dimers from monomers before hTRiC is fully dissociated.

**Supplementary Figure 5.** Uncropped gels, blots and autoradiographs.

**Supplementary Table 1**. Primers used in molecular cloning.
**Supplementary Table 2.** Parental vectors expressing CCT used in molecular cloning


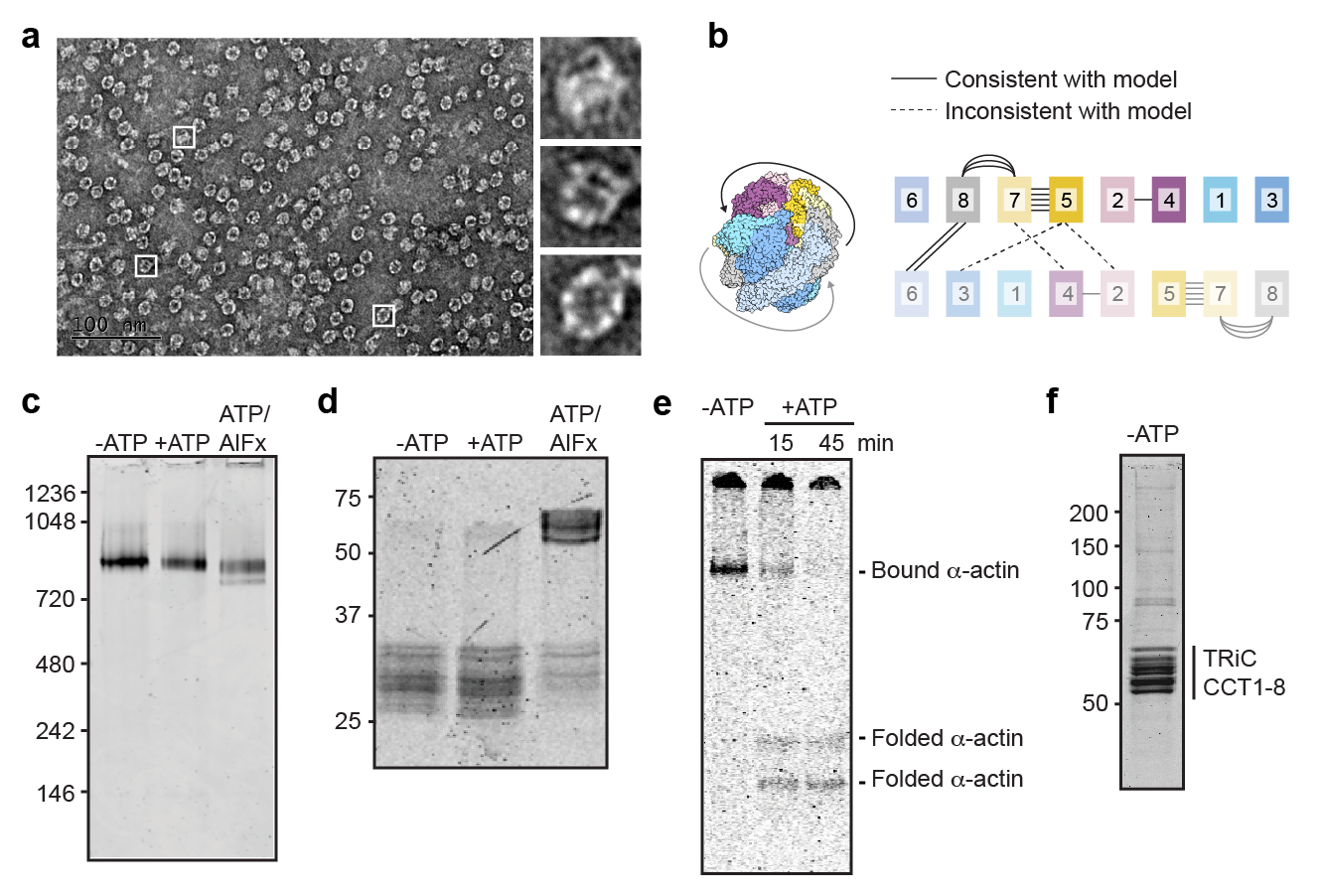


**Supplementary Figure 1.** Structural and functional validation of recombinant hTRiC. **a.** Negative stain electron microscopy reveals assembled double barrel rings. Right, detail of side and top views. **b.** Schematic of inter-subunit cross-links identified by XLMS. Coverage is in agreement with prior application of this method, with 3.9% FDR (Supplementary Data 1). Straight and curved lines denote cross-links mapped to equatorial and apical domain oligomeric interfaces, respectively. **c.** Clear native PAGE confirms the size of the complex to be approximately 1 MDa. Addition of ATP promotes lid closure and conformational cycling with no change by native PAGE. ATP/AlFx interrupts cycling by locking the complex in a closed state, resulting in slightly altered migration in the gel. **d.** Proteinase K (PK) protection assay for conformational cycling. Addition of ATP/AlFx disrupts cycling, resulting in less proteolytic cleavage. Samples analyzed by SDS-PAGE. **e.** Autoradiogram of native PAGE [35S]-actin folding assay. Actin binds hTRiC as an unfolded substrate then is folded and released upon addition of ATP. **f.** SDS-PAGE of hTRiC (CCT1-CBP). All molecular weight markers to the left of gel images are in kDa units.

**
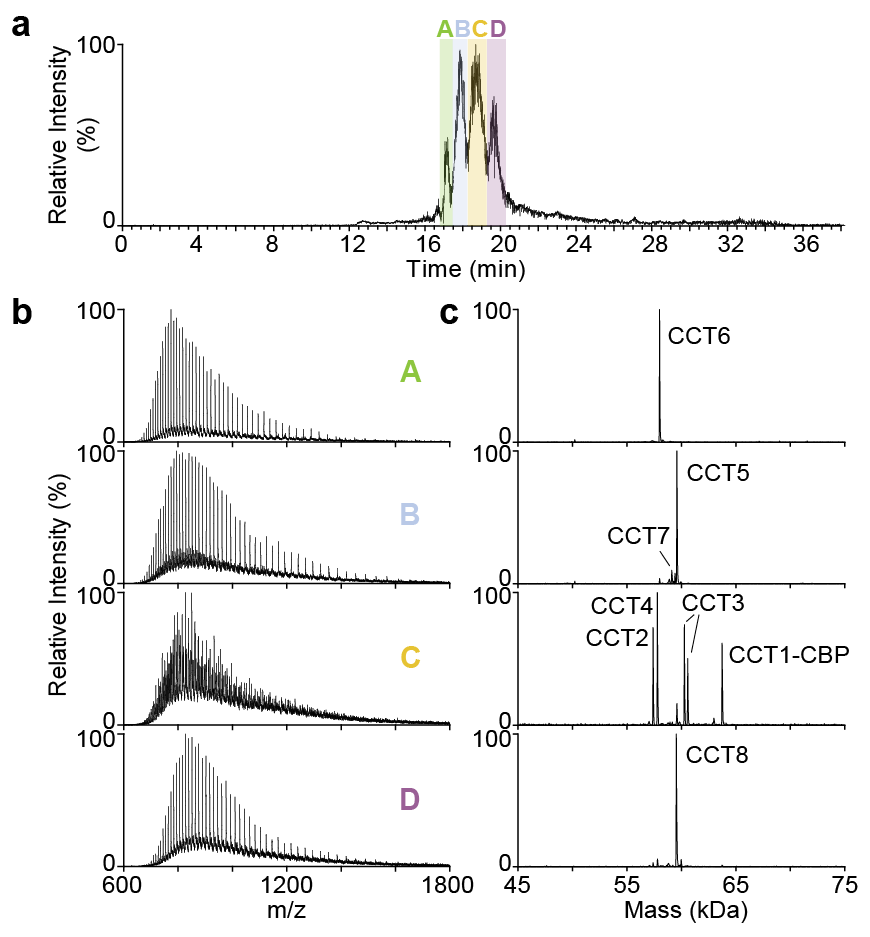
**

**Supplementary Figure 2.** Intact LC-MS of hTRiC allows orthogonal subunit mass measurement. **a.** Chromatogram of hTRiC elution from a C-18 reverse phase column. **b.** Mass spectra summations of the regions highlighted in *a*. **c.** Deconvolution of the mass spectra in *b* reveals intact masses of major species present in the sample, which match the masses of CCT subunits measured by alternative MS methods. Labels correspond to subunit assignments as described in the main text and Supplementary Data 2. The intensity of the majority-CCT5 elution peak *B* is roughly equivalent to that of peak *C* which contains four subunits, evidencing the relative stability of CCT5 following denaturation of hTRiC.

**
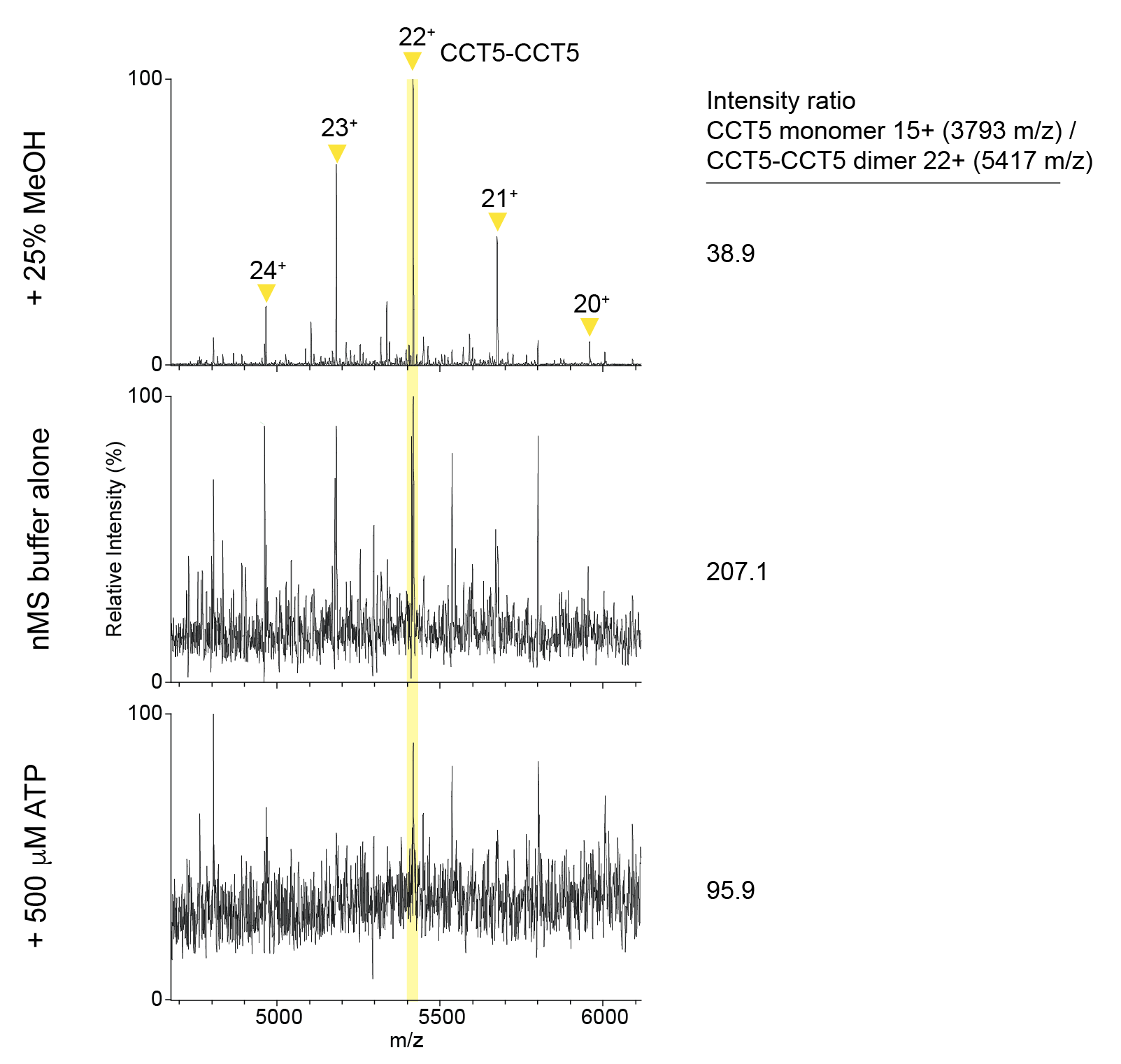
**

**Supplementary Figure 3.** Dimers begin to form from monomers before the hTRiC complex is fully dissociated by addition of organic solvent. The CCT5-CCT5 22+ peak (yellow), prominent in native mass spectra after addition of methanol (top), is also present in the native MS buffer alone (middle) and is present but especially weak after the addition of ATP (bottom), supporting ATP-driven stabilization of the complex. Absolute intensity ratios (right) are roughly proportional to monomer:dimer concentration ratios.


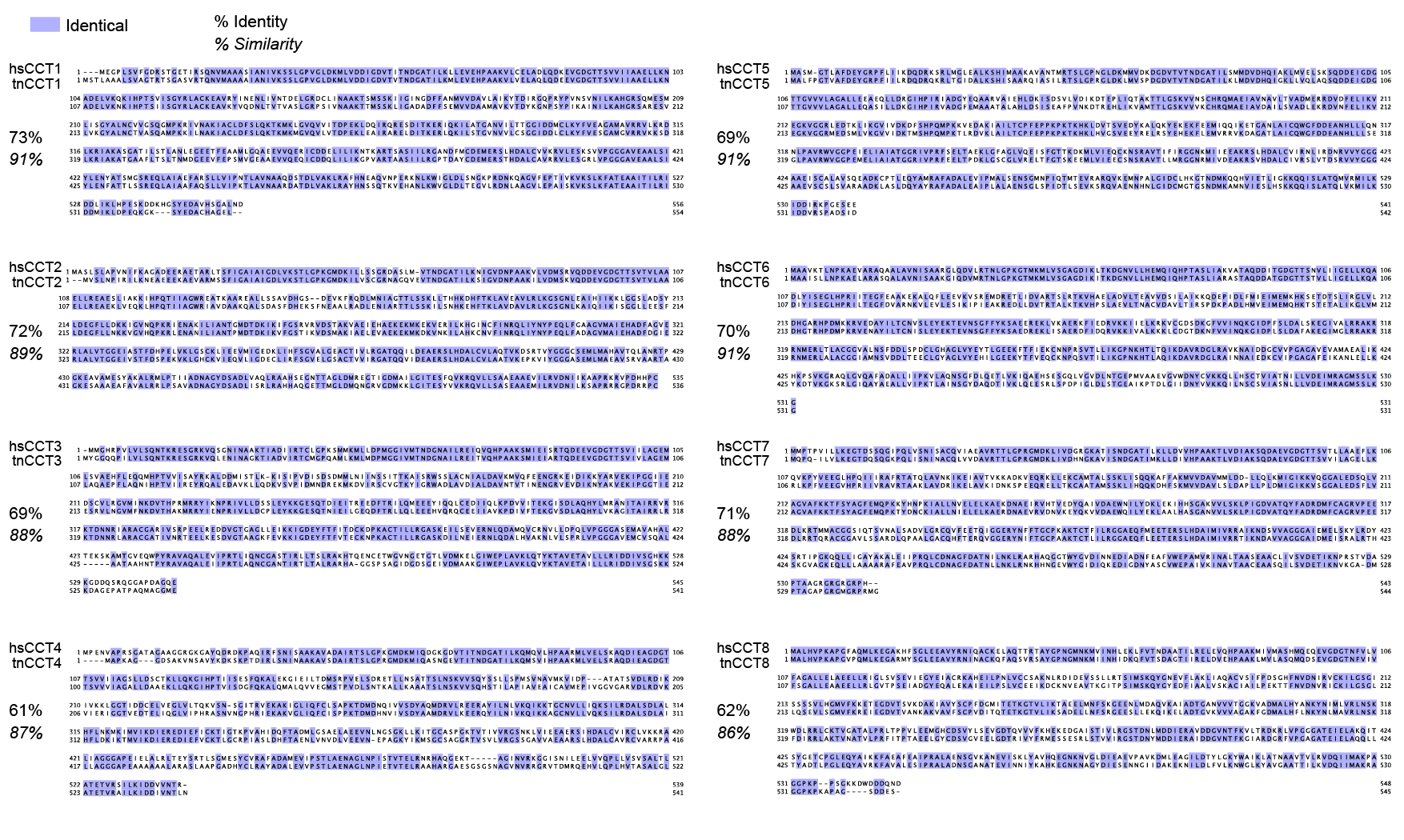


**Supplementary Figure 4.** Pairwise sequence alignments of *H. sapiens* and *T. ni* CCT subunits. Gaps are indicated by dashes, and residue positions in color are identical within that alignment. Numbers to the left of each alignment denote percentage identities (top) and similarities (italicized). Alignments were generated using Muscle v3.8.31[^1^](https://paperpile.com/c/a4bnJT/foS1) and visualized in JalView.[^2^](https://paperpile.com/c/a4bnJT/Mhp5)

**
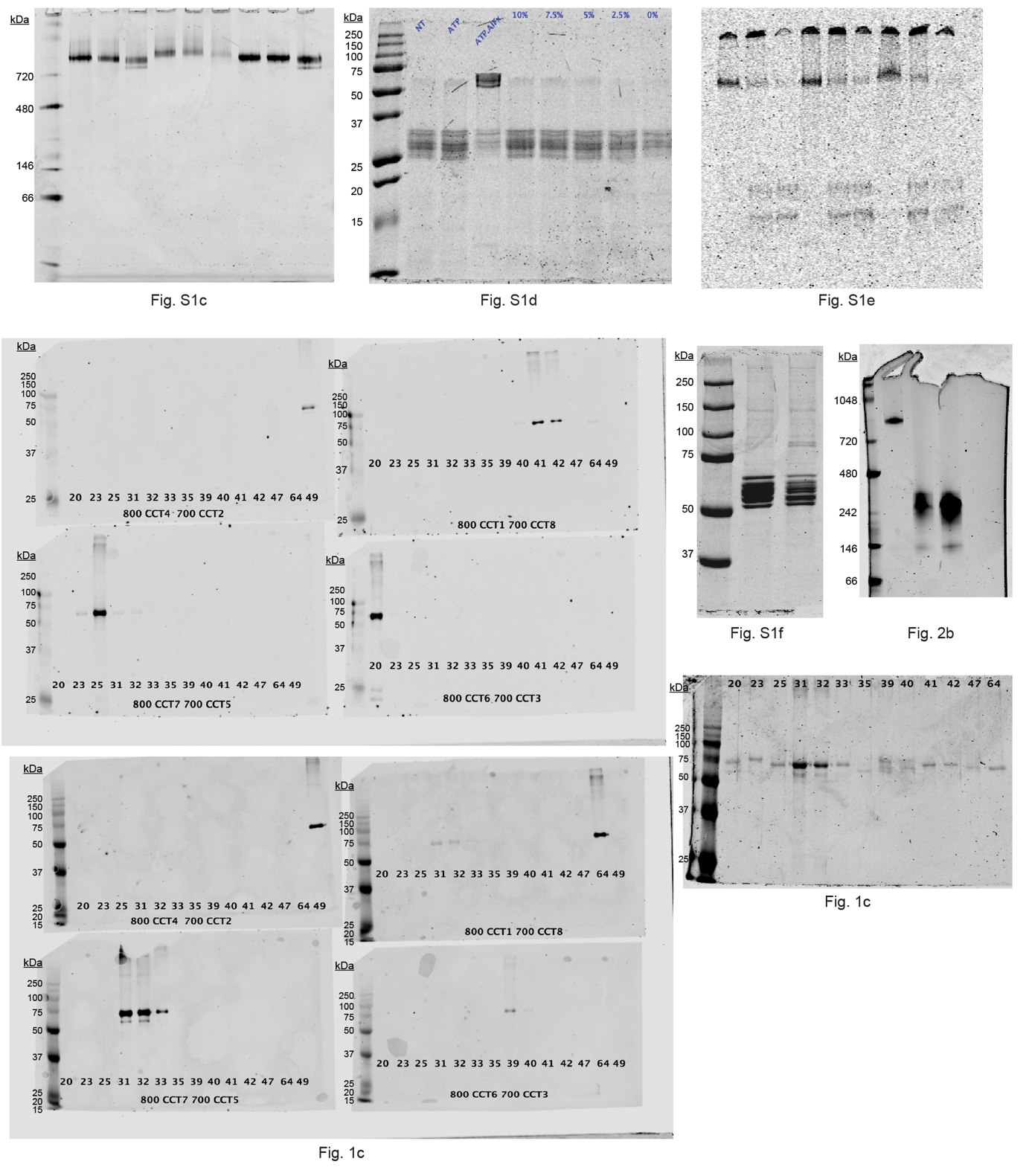
**

**Supplementary Figure 5.** Uncropped electrophoretic gels, Western blots, and autoradiographs shown elsewhere in this study. Labels under gels correspond to the locations of cropped versions. Molecular weight markers are noted to the left of each image where available.

**References**

1. [Edgar, R. C. MUSCLE: a multiple sequence alignment method with reduced time and space complexity. *BMC Bioinformatics* **5**, 113 (2004).](http://paperpile.com/b/a4bnJT/foS1)

2. [Waterhouse, A. M., Procter, J. B., Martin, D. M. A., Clamp, M. & Barton, G. J. Jalview Version 2--a multiple sequence alignment editor and analysis workbench. *Bioinformatics* vol. 25 1189–1191 (2009).](http://paperpile.com/b/a4bnJT/Mhp5)
