# Supplementary material for: Native mass spectrometry analyses of chaperonin complex TRiC/CCT reveal subunit N-terminal processing and re-association patterns
Source: Sci Rep. 2021 Jun 22;11:13084. doi: 10.1038/s41598-021-91086-6 (PMC8219831; doi:10.1038/s41598-021-91086-6)
Supplement: Supplementary file 2 — Supplementary Information 2. [file 41598_2021_91086_MOESM2_ESM.docx]

**Supplementary Table 1**. Primers used in molecular cloning.

| **Name** | **Sequence** | **CCT amplified** |
| --- | --- | --- |
| YC64 | AACGCTCTATGGTCTAAAGATTTAAATGGAATATTAATAGATCATGGAGATAATTAAAATG | CCT1-CBP, CCT1, CCT2 |
| YC65 | AAACGTGCAATAGTATCCAGTTTATTTAAATCCTAGGCTCAAGCAGTGATC | CCT1-CBP, CCT1, CCT2 |
| YC66 | AAACTGGATACTATTGCACGTTTAAATGGAGTATACGGACCTTTAATTCAAC | CCT8, CCT4 |
| YC67 | AAACATCAGGCATCATTAGGTTTATTTAAATCGAACCCCGCGTTTATG | CCT8, CCT4 |
| YC68 | AAACCTAATGATGCCTGATGTTTAAATGGAATATTAATAGATCATGGAGATAATTAA | CCT6, CCT7, CCT7-6×His |
| YC69 | AAACTAAGCTATGTGAACCGTTTATTTAAATCCTAGGCTCAAGCAGTGATC | CCT6, CCT7, CCT7-6×His |
| YC70 | AAACGGTTCACATAGCTTAGTTTAAATGGAGTATACGGACCTTTAATTCAAC | CCT3, CCT5 |
| YC71 | AACCCCGATTGAGATATAGATTTATTTAAATCGAACCCCGCGTTTATG | CCT3, CCT5 |
