# Supplementary material for: Native mass spectrometry analyses of chaperonin complex TRiC/CCT reveal subunit N-terminal processing and re-association patterns
Source: Sci Rep. 2021 Jun 22;11:13084. doi: 10.1038/s41598-021-91086-6 (PMC8219831; doi:10.1038/s41598-021-91086-6)
Supplement: Supplementary file 3 — Supplementary Information 3. [file 41598_2021_91086_MOESM3_ESM.docx]

**Supplementary Table 2.** Parental vectors expressing CCT used in molecular cloning.

| **Name** | **Description** |
| --- | --- |
| pYC12 | pFastbac Dual-CCT8, CCT1-CBP |
| pDG446 | pFastbac Dual-CCT8, CCT1 |
| pDG445 | pFastbac Dual-CCT3, CCT6 |
| pDG443 | pFastbac Dual-CCT4, CCT2 |
| pDG444 | pFastbac Dual-CCT5, CCT7 |
| pDG463 | pFastbac Dual-CCT5, CCT7-6×His |
